# Enhancing resilience of nursing education during war: policy implications from a qualitative study

**DOI:** 10.1186/s13584-025-00732-1

**Published:** 2025-11-17

**Authors:** Ilya Kagan, Odeya Cohen

**Affiliations:** 1 Nursing Department, Ashkelon Academic College, Ashkelon, Israel; 2 Department of Nursing, Recanati School of Community Health Professions, Faculty of Health Sciences, Ben Gurion University, P. O Box 653, Beer-Sheva, 8410501 Israel

**Keywords:** War, Emergency, Nursing academy, Nursing faculty, Policy implications, Resilience

## Abstract

**Background:**

Education of nurses may be compromised during a crisis. On October 7, 2023, the “Iron Swords” war broke out in Israel. The academic year was postponed, and nursing education was disrupted. Despite the acknowledged importance of continuing nurse training during emergencies, research on this topic is scarce.

**Aim:**

To examine nursing school management activities and describe the adaptive strategies of academic frameworks during the war in Israel.

**Design and methods:**

This qualitative study was conducted in nursing schools in Israel that were directly affected by the Iron Swords War (October 7, 2023). We interviewed five of the seven chairs of nursing departments in Iron Swords conflict zones, content analyzed the interview text, and employed COREQ reporting guidelines for this qualitative study.

**Results:**

Six main themes were identified: Management and leadership under uncertainty; Resilience and preparedness; Supporting well-being and inclusivity; Adaptability and innovation in the educational process; Fostering a sense of community; and Engagement of students and faculties in the crisis response. One of the most remarkable findings was the blending of personal and professional roles, as manifested in the immediate response and re-organization by the academic and administrative staff.

**Discussion:**

The ability of academia to maintain function and make decisions under conditions of uncertainty is of paramount importance. This requires collaborative decision-making, leveraging diverse perspectives, fostering inclusivity, and enhancing the effectiveness of responses. Maintaining routines creates a feeling of belonging, promotes setting and realization of goals, provides meaning, and maintains motivation.

**Conclusions:**

The results provide insights into the management of nursing education during prolonged emergencies and health policy implications. Although focused on a war scenario, the findings have broader implications and offer strategies applicable to more general crises.

## Background

Nurses strive to ensure equitable and safe care for all populations [1, 2]. However this aim may be challenged during crises and the many negative consequences for the health service. Beyond the heavy workload and treatment burden, there may be significant consequences for the functioning of the system due to direct damage to hospitals and community health services, and physical and mental harm to medical staff. This may manifest as patients with traumatic injuries, damage to health institutions and organizations, destruction of treatment infrastructure, and serious disruption to the ability to educate and train caregivers in the healthcare professionals [3–5]. In an era of global nursing shortage, special attention is required to ensure the sustainability of healthcare organizations and maintain continued functioning of the healthcare system during emergencies, such as natural disasters and wars. Reports of increased burnout and dropout of nurses due to workload and exposure to traumatic experiences during the COVID-19 [6], or treating the seriously wounded in war [7], raise the need for urgent preparations to recruit additional nurses for hospital and community healthcare.

The International Council of Nurses (ICN) has outlined eight key domains of disaster nursing competencies, intended for application across various levels, from clinical practice to policy development [8, 9]. Nurses play a critical role as leaders, educators, responders, policymakers, and researchers in disaster preparations and responses, as well as in maintaining peace and public health [10]. Accordingly, military or civilian nurses provide direct and indirect care and support to individuals, communities, and populations affected by emergencies and wars. Agaoglu et al. (2024) examined coping, taking responsibility, and decision-making among 563 hospital nurses in Hatay Province, southeast of Turkey, following a severe earthquake that resulted in massive destruction [11]. Researchers reported that emergency response training helped nurses make focused decisions and demonstrate effective behaviors while responding to an emergency. However, crisis leadership and the provision of care in emergencies may come at a cost. Mani, Innab [12] reported that nurses prioritize direct clinical care over strategic planning and long-term recovery, and are at a higher risk of suffering negative consequences than physicians [13]. In addition, there are a number of literature reports of disruptions, challenges, and traumatic experiences among healthcare workers and nurses under fire and in times of war [14, 15].

### Nursing education in times of crisis

One of the central tenets of nursing practice is the education of future generations of nurses while preserving and assimilating the profession’s values. However, this may be disrupted by gaps in planning and training that compromise the ability to respond effectively to emergencies such as pandemics, natural disasters, and war [10]. Substantial changes have occurred, and much experience has been gathered, following the COVID-19 pandemic, with consequent redesign of nursing curricula, and the establishment of partnerships to strengthen student clinical experiences, and address the needs of the community [16]. A meta-analysis of 56 studies that examined barriers and challenges to the education of health professionals during wartime in 17 countries noted five key areas of concern [4]: the need for changes in the curriculum in order to adapt to wartime constraints; lack of resources for clinical field training; the availability and competence of educators (teaching staff, practitioners, and clinical instructors); quality of life, and post-traumatic experiences among those involved in the educational process; and administrative and policy frameworks related to the training of health professionals. It is essential to note that, most of the cited studies focused on medical students and residents, with only approximately 10% of those surveyed being nursing students. The literature on nursing education during times of war or in crises is scarce and we were not able to find publications or empirical studies concerning the management of nursing educational systems in disaster or war situations.

### The iron swords war

On October 7, 2023, Israel was attacked by Hamas from the Gaza Strip in the largest terror act in the history of Israel killing over 1,200 civilians, IDF soldiers, and foreign nationals, and wounding more than 5,000 people. An additional 203 hostages were abducted to Gaza, and over 7,000 missiles were launched, leading to civilian casualties and extensive property damage. This instigated the “Iron Swords” war [17, 18] that was still continuing during the preparation of this manuscript. In the following period, additional fronts opened when Israel was attacked by Hezbollah in the north, ballistic missile from Iran, separatist groups from Iraq and the Houthis from Yemen.

At the time of the event itself, there were reports of numerous incidents of extraordinary professional care and coping by nurses who found themselves in combat zones. Nurses living on the border provided care to the wounded, under fire, with no or limited medical resources. Segev et al. (2024) analyzed qualitative data from video-recorded testimonies given by nurses and midwives from the early days of the Iron Sword War in Israel, highlighting their rapid understanding of essential steps and activities, as well as their personal and professional strength and creative decision-making in this life-threatening situation [19].

The first months of the war were challenging and accompanied by heavy shelling of civilian settlements. Life in southern Israel was disrupted and halted. Civilian services, including and academic institutions, were partially or completely non-operational. Emergency and inpatient and community health systems worked under the unprecedented burden of treating casualties of the Hamas invasion and war wounded, as well as providing routine care to the population [17]. This situation continued as the war went on. Indeed, many Israeli residents report experiencing collective trauma following the events of October 7th and the war that continues to this day.

### Impact on nursing education

Nursing education programs in Israel are uniform and are based on the Nursing Core Curriculum determined by the licensing department in the Nursing Division [20]. This serves as the minimum standard of practice and must be studied to be recognized as eligible to take the Governmental Licensing Exam. The actual education and training are conducted in academic institutions throughout the country. There are currently 6.18 nurses in Israel per one thousand people, which is lower than the average for OECD countries [20]. Many efforts have been invested in increasing the number of nurses in Israel, including increasing the number of nursing students. Nurse training is conducted in 29 educational institutions: 14 academic nursing departments (5 in universities and 9 in academic colleges); and 15 nursing schools operating in hospitals and affiliated with academic institutions [20]. Seven of the 14 academic departments are located in areas close to the Israel’s southern (3), northern (3), and eastern (1) borders, where the number of nurses is low compared to other areas. Seven other academic institutions are located in more inland areas closer to central Israel.

The academic year consists of three semesters. The year begins at the late of October or the beginning of November. The 2023–2024 academic year was scheduled to open on October 15, 2023, but was postponed depending on the academic institution’s policy. Academic institutions close to the conflict zones postponed the opening of studies four times, with the year opening 3 months late at the end of December 2023. The war engendered a deep crisis in academia along with essentially the entire Israeli population who were exposed to deaths and injuries.

Both natural and man-made disasters have been shown to cause posttraumatic distress, depression, anxiety symptoms, somatic responses to stress, sleep disturbance, burnout, and alcohol abuse in medical responders, with nurses at a higher risk than physicians [13]. However, there are few reports about the consequences of the “Iron Swords War”. Nurses exposed to challenging and life-threatening events and experiences continued to function and care for others [21]. Segev, Levi [22] described a project of mutual psychological support between nurses in the conflict zones and those from safer areas of the country. The study reported challenges in accompanying nurses in emergencies and the difficulty of receiving psychological support.

Despite the acknowledged importance of continuing the training of nurses during prolonged emergencies, research reports on this topic are scarce. Since evidence-based policies are needed to guide the management of nursing education during prolonged emergencies, the authors of this study chose to focus on the responses of the Chairs of nursing education departments, in order to examine the management activities and describe the strategies adopted by academic frameworks in conflict zones during the war in Israel.

## Methods

### Research design and participants

This qualitative study employed a participatory action research approach, integrating participation, action, and research in a collaborative process designed to both deepen understanding of a social issue and promote meaningful change. The study employed content analysis of interviews conducted between September and October 2024. A purposive sample was obtained (Chairs of Nursing Departments) in order to recruit participants with real-world experience. The Chairs of the nursing departments in seven academic institutions in conflict zones during the Iron Swords War in Israel were invited by e-mail and phone to participate in an interview. Five (72%) agreed to participate: three from the Southern; one from the Northern; and one from the Eastern borders of Israel.

### Data collection process

One of the authors, a senior nurse researcher)RN, PhD, female) and department Chair, brought both professional experience and prior knowledge of the nursing education system, and emergency preparedness and response. To reduce bias, the interviewers practiced reflexivity by keeping notes before and after each interview, documenting personal assumptions, and potential influences on the interview process. These reflections were consulted during the data analysis in order to enhance neutrality and transparency. The semi-structured interview questions were guided by the research aims and questions of this study (see Appendix 1). The interviewer has extensive experience in conducting personal interviews and has published several qualitative research reports. The interviews were conducted by the “Zoom” app (www.zoom.us) at a time convenient for the interviewee. Each interview lasted about 60 min and was recorded and transcribed by a transcription service. The decision to stop data collection was jointly confirmed by both authors during preliminary thematic analysis.

### Ethical considerations

The study was approved by the Institutional Review Board of the Ben Gurion University in the Negev (approval # 697-2 date: July 14, 2024). Participants were provided with detailed information about the study’s purpose and procedures, and their rights, including the right to withdraw at any time without consequences. Informed consent was obtained in writing before the interview commenced. Confidentiality and anonymity were strictly maintained. All personal data were coded; the personal details of the participants did not appear in the transcript.

### Data analysis and synthesis

The transcribed interviews were analyzed by the authors (OC and IK), who specialize in qualitative content analysis. Content analysis was conducted inductively from text units. The analysis unit was a word, phrase, or sentence related to the purpose of the study, the research question, and the investigated issues. First, the researchers looked for similarities and differences between the participants’ statements. Similar statements were classified under the same category according to their compatibility with the research topic. The collected data were then classified according to themes [23].

### Rigor

Study rigor was maintained by ensuring the trustworthiness and credibility of the data collected and analyzed [23]. Trustworthiness was upheld by the authenticity of the data collected from the Chairs of the nursing departments/schools, who were familiar with all the processes of the last year. Credibility was achieved by systematic content analysis followed by crosschecking by both authors. The validity of the interpretations was sustained using respondent validation during the interview and data analysis, enabling researchers to apply a comprehensive approach to the findings from the interviews [24]. Rigor was enhanced by a peer review of the results by both authors, an iterative process that ensured consistency and minimized bias in the interpretation of findings.

## Results

### Participants

Five Chairs of the nursing departments (RN and PhD degrees) of three academic institutions (one in the South, one in the East, and one in the North), participated in the study. The average age of the interviewed nurses was 52.4 ± 5.85, with an average seniority in nursing of 30.2 ± 6.49 years. Four were women and represented a range of leadership roles in nursing education through their professional careers, with experience ranging from 1 to 5 years in their current positions. Their insights highlighted the diverse contexts of their institutions, which serve between 350 and 600 students. The participants demonstrated expertise in navigating challenges related to crisis management, clinical training, curriculum development, and student support amidst the prolonged emergency that persisted from October 7, 2023, to October 2024. Table 1 summarizes the participant characteristics.

**Table 1:** Participant characteristics

Interviewee	Age	Seniority in nursing (years)	Years in current position	Location of the institution	Number of Students
**1**	52	32	4	South	500
**2**	51	29	2	South	600
**3**	63	41	4	South	600
**4**	45	21	2	Centre	400
**5**	51	28	4	North	350

### Main themes and categories

The results identified six main themes in managing nursing education settings during war, focusing on balancing the needs of students, faculties, and the community alongside the educational requirements of this challenging period. The interviewees noted a blending of national, personal, and professional experiences connected to the October 7th attack and expressed deep concerns for their relatives, children, and community members. Gradually, their professional roles came to the forefront as they began seeking information and mapping the status of their colleagues and students.

*“Initially*,* my thoughts were entirely with my family*,* especially ensuring my kids and parents were safe. However*,* soon*,* I had to shift focus and check on the students and faculty. It was overwhelming but necessary to step into my professional role… We face unique challenges due to the regional situation and its impact on students and staff.” (1). “The immediate reaction was to protect and connect with my family. Then*,* as the days unfolded*,* I started reaching out to colleagues and students to map their situations and see who needed urgent support.” (3)*.

This situation was further intensified by the critical role of nurses and nursing students in mass casualty events. Their ability to support the healthcare system and address national needs was evident. The participants’ roles were described as serving as a bridge between national agencies and acting as a pillar of resources to strengthen the system. The first phase focused on mapping the needs and circumstances of students and faculties.

*“We had to assess the preparedness of our faculty and students*,* especially in light of recent emergencies.” (4)​. “Our school has been focusing on identifying and addressing the unique needs of students in the northern region*,* especially given the recent events.” (5)*.

Managing the academic year amidst the conflict gradually became a critical issue, particularly in maintaining clinical training. It was important to safeguard the progression of the academic year, given the vital role of nursing and the needs of the national healthcare system. The results of our analysis, provide insights into managing nursing education during prolonged emergencies that can be generalized for international applications.

We extracted 68 codes organized into 17 categories and 6 overarching themes during content analysis. Table 2 presents the coding tree of the study analysis.

**Table 2:** Themes, categories, and codes - managing nursing education during war

#	Theme	Category	No. of Codes
1.	Management and leadership under conditions of uncertainty	Decision-making in crises	5
		Crisis communication	4
		Identifying physical and mental health needs	5
		Resource allocation	5
5.	Resilience and preparedness	Emergency preparedness	4
		Logistical challenges	4
		Addressing diverse needs	3
8.	Supporting well-being, and inclusivity	Mental health and emotional support	4
		Inclusivity initiatives	3
10.	Adaptability and innovation in the educational process	Institutional flexibility	4
		Creative problem solving	4
		Rapid integration of technology	4
		Scalable innovations	4
14.	Fostering a sense of community	Community-building initiatives	3
		Collective support	6
16.	Student and faculties engagement in crisis response	Volunteering in healthcare	2
		Support for emergency services	4
**Total**	6 themes	17 categories	68

### Management and leadership under conditions of uncertainty

This theme demonstrates the adaptive strategies used by nursing school leaders to respond effectively to crises. Collaborative decision-making, which involved faculty meetings and forming rapid response task forces, was critical. Participant (1) shared, *“We organized emergency faculty meetings and established a rapid response task force to address the ongoing uncertainties”*. Additionally, scenario-based planning was used to anticipate challenges and devise flexible strategies to manage academic and operational needs.

Crisis communication was another key component, with participants leveraging tools such as WhatsApp, Zoom, and email to ensure real-time updates and address misinformation proactively in order to minimize confusion during rapidly evolving situations. *“Real-time updates via WhatsApp became essential to ensure consistent messaging and to address misinformation”* (4)​.

Mapping physical and mental health needs involved preparing health status spreadsheets, tracking absenteeism due to mental health challenges, and identifying signs of faculty burnout. *“Tracking absenteeism due to mental health was a priority to ensure our faculty’s and student’s well-being”* (5)​. These efforts provided a structured way to identify, monitor, and address individual needs.

Lastly, resource allocation emerged as a significant challenge. Leaders, reallocated budgets to prioritize mental health support, and temporarily redistributed staff to ensure continuity. One participant explained, *“We had to reallocate funds for mental health support and streamline emergency budgets to meet immediate needs”* (3)​.

### Resilience and preparedness

This theme highlights the proactive measures and adaptive strategies employed to sustain nursing school operations and ensure student and faculty well-being. The essential emergency preparedness involved the development of contingency plans, safety drills, and evacuation protocols. *“Emergency preparedness included developing protocols for evacuations and conducting safety drills*,* which provided clarity and confidence during uncertain times”* (3).

The logistical challenges raised the need to adjust clinical placements, renegotiate hospital agreements, and address transportation disruptions, to meet academic requirements and operational challenges. *“We had to adjust clinical placements and renegotiate agreements with hospitals to maintain continuity in training”* (5).

Addressing diverse needs involved supporting marginalized students, including those from Bedouin and Arab communities. Measures included ensuring internet connectivity and adapting resources for students from underserved communities or those with special needs following exposure to trauma or evacuation. A participant explained, “*We made adjustments to meet the unique needs of students from diverse backgrounds*,* recognizing that emergencies often exacerbate existing disparities*” (3).

### Supporting well-being, and inclusivity

This theme focuses on initiatives designed to prioritize mental health and inclusivity during prolonged emergencies, beyond the initial mapping of faculty and students conducted at the beginning of the crisis. Mental Health and Emotional Support relates to targeted interventions for students and faculties, including mental health task forces, counseling services, and direct communication. For instance, a participant noted, *“We established a system of outreach where faculty members called individual students to maintain personal contact and address their concerns”* (1)​. Transparency and communication were also vital during clinical training. Another participant explained, *“I held open Zoom sessions with students to navigate sensitive conversations and maintain emotional support”* (5)​.

The category of Inclusivity Initiatives relates to efforts made to ensure that all community members felt valued and respected. Activities included intercultural celebrations and enforcing a zero-tolerance policy for discrimination. A participant elaborated, *“We organized joint cultural celebrations for Jewish and Muslim holidays*,* fostering mutual respect and understanding”* (3)​. Additionally, cultural competency training sessions were implemented to educate staff and students about diversity in professional and academic settings.

### Adaptability and innovation in the educational process

The theme of Adaptability and Innovation relates to the flexible and innovative strategies adopted by nursing education to address the challenges of prolonged emergencies. Institutional Flexibility was pivotal, with schools introducing hybrid-teaching models, flexible clinical schedules, and adjusted academic deadlines. One participant noted, *“We adapted clinical placements into blocks*,* enabling students to progress despite disruptions”* (2). Another emphasized, *“We implemented high-tech hybrid classrooms during the crisis*,* which proved invaluable for maintaining learning continuity”* (3).

Creative Problem Solving involved adapting lab simulations for online platforms, allowing students to continue their training remotely. “*We designed simulations that could be conducted online*,* enabling students to practice clinical scenarios remotely*,” explained one participant (5). The Rapid Integration of Technology sustained operations by enabling task-sharing, digital clinical training, and instant communication through online platforms. One participant noted, *“We quickly moved administrative and teaching operations to online platforms*,* which allowed us to operate seamlessly during the crisis”* (1).

Scalable Innovations ensured the longevity of these practices. Nursing schools institutionalized hybrid teaching models and expanded virtual mental health programs, which participants described as critical for building resilience. *“The hybrid modules we adopted are now part of our standard practices and have been critical for building future resilience”* (4).

### Fostering a sense of community

This theme reflects how operational strategies during emergencies inadvertently created a strong sense of connection between students and faculty as a result of efforts to maintain continuity and provide support. Participants emphasized the therapeutic value of outreach, with one noting, *“I personally called around 150 students*,* and just creating this contact was therapeutic for them. Many expressed gratitude*,* and it was moving to see the impact”* (1, 2)​. Virtual group activities and meetings further reinforced this unintentional outcome, by offering opportunities to connect and share experiences.

Collective Support emerged through peer mentoring and faculty-student forums, which became platforms for shared coping strategies and emotional resilience. A participant noted, *“We organized weekly Zoom meetings for each academic track*,* which became spaces to share challenges and support each other”* (3)​. Emotional resilience workshops also played a role, by offering practical strategies to navigate the crisis: *“We created emotional support workshops to help faculty and students manage their stress and maintain balance during these tough times”* (5)​.

### Student and faculty engagement in crisis response

The theme of Student and Faculty engagement in Crisis Response relates to the proactive roles played by nursing students and faculty in supporting healthcare and emergency operations during the crisis. Immediately upon the outbreak of the war, students and faculty members who requested to volunteer were invited by hospitals and clinics in the community to assist during times of need. They were then assigned tasks according to their level of training. One participant shared, *“Our students volunteered at three hospitals helping with tasks such as comforting patients and supporting nursing staff”* (1)​. Another participant emphasized: *“We trained our students in blood sampling and vein access to ensure they were well-prepared to assist during the crisis”* (5)​.

Nursing schools also coordinated efforts with national agencies and community organizations. A participant (4) noted: *“We responded to requests*,* dispatching students to assist with logistics and providing assistance in the community”*. Faculty members also initiated collaborations with other institutions to maximize the impact of their students’ contributions. One participant highlighted, *“We maintained close connections with hospitals*,* ensuring our students had meaningful roles in emergency responses. In addition*,* several of our faculty members served as nurses in evacuated communities”* (2)​.

## Discussion

This study was designed to investigate how the nursing education system adapted to maintain function in conflict zones during the first year of the Iron Sword war in Israel. The results reveal six main themes that provide a rich picture of academia under fire and the solutions proposed to deal with a prolonged emergency. Our findings are related to the Self-determination theory (SDT), developed by Deci and Ryan [25], which focuses on the role of intrinsic motivation and the fulfillment of three fundamental psychological needs for promoting well-being and optimal functioning, namely autonomy, competence, and relatedness. When these needs are met, individuals are more likely to exhibit intrinsic motivation, and enhanced engagement and satisfaction [25].

Most studies that have discussed this issue were conducted during the COVID-19 pandemic. However, while acknowledging the importance of flexibility and scalability in maintaining academic progression, there was less reference to the integration of clinical preparedness [26]. Other studies provided an overview of Faculty-led support strategies for students, particularly in clinical fields, but lacked comprehensive emergency management frameworks [27].

One of the most remarkable impressions that runs through all the interviews is the blending of personal and professional roles, in response to the deeply traumatic events at the beginning of the war, as manifested in the immediate response and re-organization of the academic and administrative staff to deal with the situation. Despite the psychosocial challenges, within a few days, steps were taken to locate and map the status of the students and Faculty members and to search for solutions to address the issues. All interviewees described mutual support for students and colleagues. Despite the risks, concerns for relatives and friends, and disruptions in all areas of life, the teams continued to function and fulfill their roles. At the same time, students volunteered for tasks both inside and outside the healthcare system. This behavior in academic nursing was consistent and similar in terms of speed and efficiency to the immediate responses of the medical and nursing teams in hospitals and community health services following the October 7th event and the outbreak of the war [17, 21, 22]. In our opinion, the similarities of the strong value base, training, and clinical background of academic and clinical practitioners dictate the level of functioning and productivity in an emergency. This may also reflect the robust role of professional identity of nurses in crises [28].

Our results emphasize the importance of the ability to function and make decisions under pressure as well as the critical role of collaborative decision-making in leveraging diverse perspectives, fostering inclusivity, and enhancing the effectiveness of responses [29]. The attempt to maintain routines, amid the chaos of an emergency, creates a feeling of belonging, facilitates the setting and realization of goals, provides meaning, and maintains motivation.

Transparent communication took on special significance in this situation, both for transferring information and updating and for support and emotional guidance. Means of disseminating information included digital applications, online and face-to-face meetings, and a selection of dedicated telephone-based systems depending on the target population. Interpersonal communication skills were critical and were used to support, guide, and direct students, colleagues, and subordinates.

Evaluating the needs of students and faculty was essential for assessing the primary consequences, planning short- and long-term interventions, allocating resources, monitoring and rehabilitating the victims, evaluating the effectiveness of interventions, and achieving goals. Identifying specific populations at risk, possibly due to language and communication problems, cultural and religious restrictions, or economic difficulties is particularly important [30]. Addressing all requests from all sectors, including minority groups, can ensure that inclusivity and equality are maintained, even in times of emergency. In preparation for future emergencies, we recommend establishing mechanisms and tools for quickly locating and identifying the needs of all involved. Preparing in advance will allow the process to be managed effectively using appropriate technologies.

Concerning the educational process, we support previous notes of the importance of flexibility in managing learning processes and readiness for frequent and unexpected changes at all levels of academic and administrative activity on the campus [26]. This includes readiness to change the curriculum, to cancel or reschedule placements, to respond rapidly to requests, and to contain frustrations, stress, and negative emotions resulting from pressure.

From a summary of the last two themes, it becomes clear that improving relationships between students and academic and administrative staff mitigates the challenges of maintaining nursing education under fire. The essential role of community in enhancing resiliency during emergencies seen here is already well established in the literature [31]. Increasing the involvement of students and faculty in decision-making regarding the educational process, may increase commitment, provide the motivation to continue to succeed, and improve performance.

The last theme describes the engagement displayed by students and faculty members who volunteered for tasks in the healthcare and support emergency services. During emergencies, the balance between needs and resources is disturbed, and there is a need to manage the professional contribution of the nursing students that can be considered a valuable resource during public health crises [32].

The study presents scalable and transferable recommendations, emphasizing key areas such as management and leadership under uncertainty. These themes outline a comprehensive pathway for enhancing resilience in nursing academia and beyond, by addressing immediate needs and long-term preparedness. Findings and insights from this study can serve as a basis for improving and refining policies for dealing with emergencies in the nursing education systems.

### Health policy implications

In times of prolonged emergency, delays in training and licensing of nursing students, may hinder the healthcare system’s ability to prepare for and cope with clinical demands. It is therefore of utmost importance to ensure the continuity of these processes, even under conditions of conflict and uncertainty. The findings of this study highlight the need for comprehensive policy frameworks that address the continuity and resilience of nursing education during emergencies. The Ministry of Health, along with academic institutions, should collaborate to develop standardized protocols that support flexible clinical placements, integrated mental health services for students and faculty, and the rapid transition to hybrid or remote learning environments [16, 26]. These policies must also include mechanisms to identify and support vulnerable populations, ensuring equitable access to educational resources and emotional support during crises [30]. The active participation of nursing students and faculty in emergency responses highlights the need for regulatory clarity regarding their roles and responsibilities and the integration of these activities with their educational commitments; strengthening the professional identity of nurses as both caregivers and educators in times of crisis may enhance engagement and leadership capacity [10, 28].

In this study, we conceptualized departments of nursing as meso-level organizational entities, distinct from individual faculty members and students, yet intrinsically connected to the broader academic, nursing education, and health systems. As such, their resilience reflects the capacity of the department to adapt, reorganize, and sustain core educational and professional functions in the face of ongoing challenges. For nursing education departments in Israel, the need for robust and adaptable education in long-term emergency and war scenarios is not merely theoretical but a lived reality. Therefore, we propose the following policy recommendations for nursing education in emergencies and wartime at two organizational levels: at the level of policymakers, and the level of the academic organization and nursing department.

Policy making:

1. Establish a national policy to guide the nursing education system during long-term emergencies.
2. Foster collaboration among nursing schools to share best practices and educational resources.
3. Strengthen partnerships with healthcare organizations to support clinical placements and joint training opportunities.
4. Develop and implement a mandatory, credit-bearing core course on disaster nursing core competencies.

Nursing schools\departments

1. Maintain and regularly update emergency preparedness protocols, including designated safety zones, key personnel, and clear operational procedures.
2. Establish internal rapid-response teams to coordinate strategic decision-making and address immediate academic and ethical challenges.
3. Conduct ongoing assessments of faculty and students’ physical and mental health needs.
4. Design flexible curricula that can accommodate disruptions to clinical training and academic schedules.
5. Maintain multiple, accessible communication channels to ensure adequate information flow during crises.
6. Integrate leadership opportunities for students in peer-support programs and emergency response initiatives.
7. Promote intercultural competence and inclusive communication practices in emergency settings.
8. Appoint a dedicated coordinator to manage student placements in healthcare organizations during emergencies.
9. Provide academic credit or official recognition for student volunteering in healthcare systems during crises.

### Study limitations and recommendations for future research

The limitations of the study are inherent to qualitative research and the subjectivity of the opinions of a relatively small number of interviewees. Nevertheless, a purposive sampling strategy was employed to recruit participants with direct, real-world experience in managing nursing education during times of war. In addition, qualitative studies provide valuable information, while the validation techniques improve the accurate representation of the studied phenomena [23, 24]. Moreover, the five Chairs of the nursing departments interviewed represent 72% of leading nursing departments in the conflict zones in Israel. Notably, we emphasized confidentiality and voluntary participation and maintained reflexive notes throughout data collection and analysis in order to overcome potential bias introduced by the dual role of researcher and department Chair. A final point is that the interviews were conducted via Zoom rather than in person. While this mode of communication enabled participation from diverse locations and ensured safety and convenience, it may have limited the observation of nonverbal cues and the depth of interpersonal connection that can occur in face-to-face settings. Nevertheless, the online format allowed flexibility and comfort, especially in times of crisis. Future studies could expand on this work by including multiple institutions to capture broader cultural and organizational perspectives. Additionally, mixed-method or longitudinal designs could be employed to examine changes over time. Further exploration of faculties and real world student experiences could deepen understanding of the any influential factors.

## Conclusions

Our results provide critical insights and policy implications into sustained maintenance of nursing education during prolonged emergencies in conflict zones. While the study focused on a war scenario, the findings have broader implications for managing nursing education during more generalized crises. This aligns with the principles of the all-hazard approach, which acknowledges that a variety of hazards challenge the health system in similar ways. Thus, emergency preparedness and response actions are usually implemented using the same model, regardless of cause [33]. By adopting such a framework, academic nursing institutions can prepare and enhance their response capacities, to ensure continuity of education and support for the health system.

## Data Availability

Data supporting the findings of this study will be available upon reasonable request to the corresponding author.

## Appendix 1

The interviewer introduces herself, explains about the study, and verifies consent to participate in the study.

Background questions:

- Geographic location, academic institution, level of activity during the war
- Briefly tell me about your professional experience
- What position do you currently hold? How long have you been in the position?

Directed questions:

- What is the organizational structure at your workplace/department?
- How many employees do you manage? What is the size of the department/number of students/staff, etc.?
- How did the October 7th event and following war affect your workplace?
- Was there any harm to the staff? Students? Families? Workplace relationships?
- Did you need to make any special preparations due to the war situation?
- How and what was the nature of the relationships with the clinical fields/Ministry of Health/Nursing Division in the MOH/nursing institutions?
- What were the actions you took during the war? What was the impact, and what was the preparation in the short, medium, and long terms?
- Have you taken steps to support students or faculty? How did they respond to the situation and express distress?
- From your experience, is there anything you recommend changing and to improve following this emergency (In practical to policy levels)?
- What are the challenges and lessons learnt from this war period?
- Do you have any specific recommendations for health policy regarding nursing education to cope with an emergency?
- Would you like to add or discuss any additional issues?

## Declarations

### Reporting guidelines

COREQ reporting guidelines were used.

### Supporting information

Appendix 1 contains the semi-structured interview framework used to explore participants’ experiences and perspectives.
